# Human rights in patient care and public health—a common ground

**DOI:** 10.1186/s40985-017-0075-2

**Published:** 2017-12-20

**Authors:** Maya Peled-Raz

**Affiliations:** School of Public Health, University of Haifa School of Public Health, Haifa, Israel

**Keywords:** Human rights, Patient care, Human rights in patient care, Public health law, Patients’ rights, Medical law

## Abstract

Medical law and public health law have both served extensively as instruments of health protection and promotion—yet both are limited in their effect and scope and do not sufficiently cover nor supply a remedy to systematic, rather than anecdotal, mistreatments in the health care system.

A possible solution to this deficiency may be found in the human rights in patient care legal approach. The concept of human rights in patient care is a reframing of international human rights law, as well as constitutional thought and tools, into a coherent approach aimed at the protection and furthering of both personal and communal health. It applies human rights discourse and human rights law onto the patient care setting while moving away from the narrow consumeristic view of health care delivery.

By applying human rights in patient care approach, both national and international courts may and should serve as policy influencing instruments, protecting the rights of the most vulnerable and prejudiced against groups, which are want of a remedy through traditional patients’ rights legal schemes.

## Background

The concept of human rights in patient care (HRPC) is rooted in the health and human rights framework and builds on the last 2 decades of work by the health and human rights movement [1].

It is a reframing of international human rights law, as well as constitutional thought and tools, into a coherent approach aimed at the protection and furthering of both personal and communal health. It attempts through the theoretical and practical application of general human rights principles to the patient care context, particularly to interactions between patients and providers ([2] at p. 7).

HRPC is viewed as a principled alternative to the growing discourse of “patients’ rights” that has evolved in response to widespread and severe human rights violations in health settings ([2] at p. 13–15).

In this paper, I would like to contend that HRPC is a conceptual link between medical law^1^and its patients’ rights subpart and public health law.

While an in-depth discussion into the similarities and differences between HRPC and patients’ rights discourse is due (and has been done elsewhere) [2], this paper will focus on the correlation between HRPC and the public health legal framework, while limiting the discussion of the former topic to only central short observations.

### What are human rights?

Ethicists use the term “human rights” to describe unchallengeable, fundamental rights to which a person is inherently entitled simply because she or he is a human being. They are commonly seen as based on natural law, which is a norm that exists independently, regardless of the law enacted by people under a certain regime, society, or country. The existence of such rights derives from philosophical reasoning and argumentation, deducing binding rules of moral behavior, via the use of reason, to analyze both social and personal human nature [3].

Human rights, through the lens of natural law theory, are aspirational in nature and are commonly (though contestable) thought to be universal and timeless.

Legal scholars, on the other hand, use the concept of human rights to refer to a body of international law that originated in response to jarring offenses against human dignity committed during World War II [4]—as well as to its derivatives and complimenting arrangements on the regional and national levels. The concept of human rights, in this context, relies on texts and precedents and is enforced through treaty obligations, as well as constitutional ones.

The main international source of human rights law is the International Bill of Human Rights, comprising the United Nations Charter, the Universal Declaration of Human Rights, and two international human rights conventions—the International Covenant on Civil and Political Rights (ICCPR) and the International Covenant on Economic, Social and Cultural Rights (ICESCR) (both adopted in 1966 and entered into force in 1976).

In its preamble, the United Nations Charter articulates the international community’s determination “to reaffirm faith in fundamental human rights, [and] in the dignity and worth of the human person.” The Charter, as a binding treaty, pledges member states to promote universal respect for, and observance of, human rights and fundamental freedoms for all, without distinction as to race, sex, language, or religion [4].

Over the years, states have created an extensive array of international conventions, declarations, and organizational frameworks to protect human rights. These conventions reflect the human rights norms that the signatory states undertake to meet and guarantee. In America,^2^ Europe,^3^ and Africa^4^ states established separate regional systems to promote and protect human rights, which include enforcement mechanisms.

In order to fulfill their treaty obligations, as well as independently of that, many states have incorporated a human rights protection scheme into their national constitutional and regulatory frameworks—articulating their tailored commitment to the furthering and protection of human rights within their borders.

### Laying the ground: medical law and public health law

Patient care and public health are two complementary and interrelated approaches for promoting and protecting health. Yet, patient care and public health can and also must be differentiated because in several important ways, they are not the same [5].

Patient care refers to the prevention, treatment, and management of illness and the preservation of physical and mental well-being through services offered by health professionals (or non-professionals under their supervision) [6]. Conversely, public health, as per the Institute of Medicine, “is what we, as a society, do collectively to assure the conditions for people to be healthy.” [7]. The fundamental difference involves the population emphasis of public health, which contrasts with the essentially individual focus of medical care. Public health identifies and measures threats to the health of populations, develops governmental policies in response to these concerns, and seeks to assure certain health and related services. In contrast, patient care focuses on individuals—diagnosis, treatment, relief of suffering, and rehabilitation [5].

Medical law (along with its patients’ rights subpart) and public health law have both long been used to protect and further human rights: the first through attempting to regulate professional conduct in patient care and the second by attempting to regulate public health engagements.

#### The patients’ rights legal approach

The medical law governs professional conduct in the patient care sphere primarily through civil law—torts law mainly. It zeros on the interaction between patients and caretakers in the patient care setting—and specifically sets its focus on the obligations of the caretaker in that relationship. It is relevant and applicable in cases where the individual—receiving or seeking treatment—suffers direct harm, due to a breach of his right to good care. Good care has been viewed to consist of not only competent and skilled practices but also of respect of patients’ rights—their ability to lead treatment decisions instead of being led through them, to maintain control of the information divulged to them and to others, to receive equal care in a non-discriminatory fashion, and to leave the health care facility when desiring so and so on.

While these rights stem directly from the fiduciary attributes of the doctor-patient relationship [8], they have gradually taken on a consumeristic form [9]. Policy analysts began to think about the potential role of patients as customers in the 1930s, in response to the rapidly rising costs of medical care ([8] at p. 586). In the 1960s, consumerist ethos has been warmly embraced by medical law as an aspect of the patients’ rights movement that challenged physician paternalism [10] and has gained additional force in the 1980s in light of the desire to shield patients from harm due to the rising need to rationalize medical expenditures [11].

Within this consumeristic framework, patients’ rights are seen as placing what resembles contractual obligations on the shoulders of health professionals—therein viewed as service providers. These obligations, when not fulfilled, may be grounds for a civil lawsuit, demanding compensation for damages—caused due to the direct violation of the right itself and/or due to physical damage that may have been caused by the said violation.

In the last 50 years, specific aspects of what good care is—and thus what a patient has a right to expect when entering the health care system—have been codified in key national and regional instruments ([2] at p. 13). These codes, along with court decisions awarding damages for breaches of patients’ rights, have gone a long way in furthering patients’ rights to good care. With that being said, key limitations must be noted to the scope of the protection rendered to human rights under the patients’ rights doctrine.

First, the patients’ rights doctrine leaves out the rights of other stakeholders in health care delivery, focusing exclusively on patients ([2] at p. 14). It does not, for example, regard caretakers’ right to safe working conditions, their freedom from (professional) coercion, nor their right to freedom of association. Second, as a paradigm that only looks at what happens inside of the patient-provider (whether a human provider or an institutional one) relationship, it is unsuitable to instances in which the cause of the infringement on human rights rests with elements which are external to the said relationship. When the (health care) system is designed—deliberately or not—in a way that is set to infringe on human rights, the patients’ rights doctrine will provide no assistance. A patient cannot claim an infringement on his right as a patient when he is not receiving equal care, due to the lack of a nationally funded health plan, nor can he protest his patient’s rights breached when a state law requires the physician to notify the authorities of his medical condition.

#### Public health law

Public health professional conduct, in contrast to the previously described patient care sphere, has long been governed by public law—through its public health law subdomain—focusing on the interaction between the state and its citizens regarding their health.

Public health law regulates the state’s authority and duty to identify and lessen community health risks as well as to promote the health of the community. At the same time, it contemplates the limits on the state’s authority to constrain the personal rights of its inhabitants—in the name of furthering the population’s health [12]. The core legal components of the public health law are the administrative, statutory, and constitutional provisions that empower or mandate a government to act for the health of the community, as well as those that bridle the state’s power to do so [12].

In many cases, human rights are intertwined with the public health law, serving both as the reasoning behind public health governmental interventions and actions, as well as a limitation on the state’s power, as it attempts to further public health goals.

WHO’s constitution states that health is the “state of complete physical, mental, and social well-being and not merely the absence of disease or infirmity”.^5^ Human rights are the basis for such conditions—headed by the right to health care, but definitely not concluding at it. People will be healthier when they get better excess to health care and when health care itself is better, but also when they themselves are better educated, when their bodily integrity is not threatened by external preventable harms, when they have a right to use pregnancy prevention and planned-parenthood tools, and so on. Governments are therefore responsible for enabling their populations to achieve better health through respecting, protecting, and fulfilling rights, i.e., not violating rights, preventing rights violations, and creating policies, structures, and resources that promote and enforce rights [13, 14].

At the same time, actions taken to further health-supporting rights may also infringe on the rights of some individuals, and should thus be scrutinized in accordance with human rights law and its application in public health law. Attempts to minimize the use of tobacco products may infringe on the smoking individual’s right to autonomy and free choice; immunization programs may harm individuals’ right to bodily integrity; and tuberculosis curtailment measures may limit individuals’ right to freedom of movement; these potential human rights violations must be weighed against the sought-after public health goals, using the balancing scheme incorporated through human rights into public health law—to be later explored.

### Defining HRPC and situating it in the legal landscape

For many years, the two approaches to health promotion—patient care, governed by medical law, and patient’s rights and public health, governed by public health law—were viewed as somewhat dichotomous—the one only interested with the quality of care delivered by the specific care-taker, while the other looking only at the power-play between the state and its citizens.

In this section, I will assert that HRPC is a gap-bridging approach to the promotion of both individual and community health—applying human rights discourse and human rights law onto the patient care setting while moving away from the narrow consumeristic view of health care delivery.

The HRPC approach relies on human rights law in the same way public health law often does, yet it is applicable to the patient care setting the way that patient’s rights law was thought to exclusively be. By doing so, HRPC makes it possible to coherently look at the actions and interests of all the relevant players involved in the health delivery and promotion interplay—including, but not limited to, specific patients, patient groups, health care providers, policymakers, and the community at large.

HRPC functions as a health promotion and protection tool by looking for systematic, rather than anecdotal, mistreatments by health care providers, which constitute either direct or indirect (through the violation of other rights) breaches of the right to quality care and to freedom from injury and bodily integrity.

An example of a direct systematic breach of the rights to health and bodily integrity may be found in state laws that allow for the forced feeding of prisoners [15]. An indirect systematic harm to health may be exemplified by notification laws, requiring health personnel to notify authorities about the treatment of illegal immigrants (i.e., infringement of their right to privacy and confidentiality)—causing them to avoid seeking health care.

Systematic mistreatments are the product of either an active policy decision, as in the two above examples, or of an undesirable common practice, which the state has been neglecting to attend to. Such neglect may, for example, be found in cases where the state does not intervene in order to curtail discriminatory and sub-par health care delivery to HIV-positive patients.

Systematic problems must be systemically addressed by the state, by amending policies or regulations, ensuring appropriate training, creating monitoring services, establishing opportunities for complaint and redress, and taking disciplinary measures when warranted ([2] at p. 7). Under human rights law, governments are obliged to respect, protect, and fulfill the rights contained in its signed treaties [16]. When the state neglects to act or refrains from using the tools in its possession to abolish human rights violations, not to mention when it actively puts the harmful policy into place, HRPC calls for the use of both international and national human rights law, in order to oblige the state to conform with its obligations to the protection and advancement of health.^6^

As not all mistreatments in patient care amount to a violation of human rights, the HRPC approach may be viewed as narrower in its application than patients’ rights law; yet as it is mainly based in international law, HRPC is applicable also in countries that do not have national patient’s rights laws, thus allowing for the protection of rights in patient care settings in countries where the protection of patients’ rights does not present a high priority for the policymakers.

More markedly, unlike patients’ rights law, which regards the rights of individual patients as intrinsically paramount (as consumer protection laws tend to do), HRPC recognizes that rights of no single patient are absolute. While as concerned with informed consent, confidentiality and the right to information—just to name a few central patients’ rights’ fundamental concepts—HRPC automatically recognizes the need to place limitations on rights in the health context for the sake of other interests, both communal and private. It does so by balancing the protection of such elements of good care, against the state’s obligation and choice to further the rights and interests of others—including the rights and interests of health care providers, which are finally given a voice, via HRPC.

### Balancing the rights to health and bodily integrity against other rights and interests

Both the International Covenant on Civil and Political Rights and the International Covenant on Economic, social and cultural rights state that rights protected thereof may be justifiably limited under certain conditions, except for the rights to life (art. 6); the right to freedom from torture and from cruel, inhuman, or degrading treatment or punishment (art. 7); the right to recognition as a person before the law (art.16); and the right to freedom of thought, conscience, and religion (art.18)—which the Covenant on Civil and Political Rights has proscribed any derogation of—all other rights may be limited as determined by law “only in so far as this may be compatible with the nature of these rights and solely for the purpose of promoting the general welfare in a democratic society” [ICESCR (art. 4)].

Correspondingly, almost every national constitutional guarantee of certain rights attaches limitations to the breadth of those rights in an effort to balance the interests of the individual with those of the community when certain conditions arise.

There are two types of limitation vehicles, the first of which is called a “derogation clause.” Derogation clauses allow states to breach obligations to uphold certain rights, for reasons related to war or a public emergency, while establishing the conditions under which the state may argue that such a state of affairs exists.

The second limitation vehicle, which is central to our discussion, is called a “limitation clause” (or “clawback” clause). This legal tool allows for the suspension or restriction of guaranteed rights to which they apply, under specific conditions.

These conditions form a balancing scheme allowing for the weighing of states’ powers and discretions against individuals’ human rights and for a critical look at the way states use (or do not use) their powers and discretion. This balancing scheme is inherent to both public health law and the HRPC approach. Yet, while under public health law, the question at play is whether a state may infringe upon human rights; in order to further and protect the public’s health, HRPC is employing the same legal scheme in order to explore the legitimacy of the state’s use of its powers when the aforementioned use (or lack thereof) infringes upon individuals’ health-related rights.

When ruled upon by either a national or an international court, a breach of this balancing scheme would (a) allow (and even require) the state to use its powers for the sake of furthering the health of the community, despite foreseeable harms to individual’s rights—under the public health doctrine—or (b) require the state to use (or cease from using) its powers, in order to protect individual’s health rights—under the HRPC approach.

Although the wording of the conditions that form the balancing scheme may differ from country to country, and from state to state, it is agreed upon^7^ that the following conditions are central to all limitation clauses:

- All limitation clauses shall be interpreted strictly and in favor of the individual rights at issue;
- No limitation on human rights shall be applied in an arbitrary manner;
- Every limitation imposed shall be subject to the possibility of challenge to and remedy against its abusive application;
- No limitation on individual rights shall be discriminatory in nature;
- For a limitation on individual rights to be deemed legitimate, it must
  - Respond to a pressing public or social need—assessed based on objective considerations;
  - Pursue a legitimate aim;
  - Be proportionate to that aim.
- In applying a limitation, a state shall use no more restrictive means that are required for the achievement of the purpose of the limitation.

### The application of the balancing scheme in the HRPC approach—a case study

In order to exemplify the use of the HRPC approach and the balancing scheme in its center, let us look at the following example: After falling off of the roof he was working on, a non-registered immigrant worker is rushed to the ER. He is treated for broken bones, and the physicians fear that he may suffer from a hemorrhage in his skull, and would like to hospitalize him for farther examination and surveillance. Regretfully, so the physician tells the patient, he will have to notify the authorities about the presence of an illegal immigrant in the hospital. The patient refuses hospitalization as well as divulging his identity to the staff and leaves the premise without proper treatment.

Statutory notification requirements, such as the one described here, are beyond a doubt a breach of patients’ right to privacy—which consequently infringe on the right to health care, of both patients and those who refrain from approaching the health system to begin with and do not acquire a “patient” status. Yet, as it is a state-mandated breach, patients, such as the one described above, can find no remedy for the sub-par health care they receive, via medical law and patients’ rights discourse.

Such a remedy could and should be sought, though, through HRPC tools. A petition can be brought forth in front of the national or international courts, claiming that the state is over-reaching its authorities and unduly infringing on individuals’ rights to privacy and health.

The relevant court would then be required to consider the following questions:

- Is the notification requirement pursuing a legitimate aim?
  It appears that notification requirements, regarding the illegal stay of immigrants, mainly aim to protect the rule of law and to prevent the violation of immigration laws. Indirectly, they aim to prevent “harms” which illegal immigration may cause to the community.
- Is it responding to a pressing public or social need?
  This question should be answered based on the data relevant to each country and each era. Relevant data should include the extant of illegal immigration experienced by the state, as well as the types and scope of harms caused by it to the community. These should be described in specifics, and not merely generally speculated upon.
- Is the harm to individual rights in this case proportionate to that aim?
  The more prominent the nature and the scope of the burden caused by illegal immigration, the more likely it is that the courts would see the infringement on individual rights as proportionate. Yet, as the harm to individual rights here is grave, only extreme burden should be deems proportional, and in any case, where it resolved that illegal immigration in of itself is not a real issue of concern and that the aim is mainly to protect the rule of law; it is likely that such a requirement would be struck down.
- Are there less restrictive means that may still reasonably achieve the legitimate aim?
  This, I believe, would serve as the main argument in favor of the abolition of this notification requirement. In order for the requirement to pass the courts’ scrutiny, evidence would have to be presented, showing that (1) it plays an integral part in the minimization of law-violations, in general, and illegal immigration in particular; (2) that, if abolished, all together or specifically inside the health system, these aims will greatly suffer; and (3) that there are no other less damaging legal tools, that may take their place in furthering the worthy goal of legal obedience.

In this author’s opinion, there are good chances that such a requirement would be struck down, or at least limited to non-health care venues—even in view of the current global immigration situation— dimmed un-proportional and overly intrusive.

## Conclusion

HRPC is a gap-bridging approach to the promotion of both individual and community health—applying human rights discourse and human rights law onto the patient care setting while moving away from the narrow consumeristic view of health care delivery. It functions as a health promotion and protection tool by looking for systematic, rather than anecdotal, mistreatments by health care providers—mistreatments which are the product of either an active policy decision or of an undesirable common practice—which the state has been neglecting to attend to.

By applying a HRPC approach, both national and international courts may and should serve as policy influencing instruments, protecting the rights of the most vulnerable and prejudiced against groups, which are want of a remedy through traditional patients’ rights legal schemes.

## Abbreviations

HRPC: Human rights in patient care
ICESCR: International Covenant on Economic, Social and Cultural Rights
WHO: World health organization

## Footnotes

1 Though several definitions have been suggested, medical law is referred to in this paper as “essentially concerned with the relationship between health care professionals and patients” (Kennedy I. and Grubb A. Medical Law 2000: 5).

2 American Convention on Human Rights (adopted 1969, entered into force 1978).

3 European Convention on Human Rights (adopted 1950, entered into force 1953).

4 The African Charter on Human and Peoples’ Rights (adopted 1981, entered into force 1986).

5 WHO, Constitution of the World Health Organization, adopted by the International Health Conference, New York, June 19 to July 22, 1946, and signed on 22 July 1946 by the representatives of 61 states. World Health Organization, 1946.

6 The notion of “enforcement” of treaty obligations under international law is a complex one. Both international and national courts may be petitioned and asked to rule as to a state’s violation of human rights—the right to health included. Yet, the enforced consequences of such a ruling are ambiguous and varied. Also, in many cases, the sought-after relief may be achieved just by the mere existence of international debate regarding the infringement, with no actual need for a formal court intervention. On this, see Cassel D. Does International Human Rights Law Make a Difference? International Human Rights Law in Practice, Chicago Journal of International Law 2001; 2: 121, and Koh H. H. How Is International Human Rights Law Enforced, Indiana Law Journal 1998;74(1): 1397.

7 See UN Commission on Human Rights, The Siracusa Principles on the Limitation and Derogation Provisions in the International Covenant on Civil and Political Rights, 28 September 1984, E/CN.4/1985/4.
